# Immigrants’ duration of residence and adverse birth outcomes: a population-based study

**DOI:** 10.1111/j.1471-0528.2010.02523.x

**Published:** 2010-04

**Authors:** ML Urquia, JW Frank, R Moineddin, RH Glazier

**Affiliations:** a Dalla Lana School of Public Health, University of TorontoCanada; b Centre for Research on Inner City Health, St. Michael’s HospitalToronto, Canada; c Public Health Research and Policy, University of EdinburghEdinburgh, UK; d Institute for Clinical Evaluative SciencesToronto, Canada; e Department of Family and Community Medicine, University of TorontoCanada

**Keywords:** Duration of residence, immigration, preterm birth, small for gestational age

## Abstract

*Please cite this paper as*: Urquia M, Frank J, Moineddin R, Glazier R. Immigrants’ duration of residence and adverse birth outcomes: a population-based study. BJOG 2010;117:591–601.

**Objective:**

This study aimed to examine preterm and small-for-gestational-age (SGA) births among immigrants, by duration of residence, and to compare them with the Canadian-born population.

**Design:**

Population-based cross-sectional study with retrospective assessment of immigration.

**Setting:**

Metropolitan areas of Ontario, Canada.

**Population:**

A total of 83 233 singleton newborns born to immigrant mothers and 314 237 newborns born to non-immigrant mothers.

**Methods:**

We linked a database of immigrants acquiring permanent residence in Ontario, Canada, in the period 1985–2000 with mother–infant hospital records (2002–2007). Duration of residence was measured as completed years from arrival to Canada to delivery/birth. Logistic regression models were used to estimate the effects of duration of residence with adjusted odds ratios and 95% confidence intervals. In analyses restricted to immigrants only, hierarchical models were used to account for the clustering of births into maternal countries of birth.

**Main outcome measures:**

Preterm birth (PTB) and SGA birth.

**Results:**

Recent immigrants (<5 years) had a lower risk of PTB (4.7%) than non-immigrants (6.2%), but those with ≥15 years of stay were at higher risk (7.4%). Among immigrants, a 5-year increase in Canadian residence was associated with an increase in PTB (AOR 1.14, 95% CI 1.10–1.19), but not in SGA birth (AOR 0.99, 95% CI 0.96–1.02).

**Conclusions:**

Time since migration was associated with increases in the risk of PTB, but was not associated with an increase in SGA births. Ignoring duration of residence may mask important disparities in preterm delivery between immigrants and non-immigrants, and between immigrant subgroups categorised by their duration of residence.

## Introduction

The reduction of preterm birth (PTB) represents one of the most important challenges in perinatal health. Preterm birth is a major predictor of perinatal morbidity and mortality, and is associated with childhood disabilities and adult onset of diseases.^1^,^2^ Preterm birth rates have increased over the last decade in Canada, from 7.0 per 100 live births in 1995 to 8.2 per 100 live births in 2004.^3^ Infants with fetal growth restriction are also at higher risk of perinatal mortality and long-term health consequences, even if born at term.^3^

Immigrant women contribute to more than one-fifth of all live births in the United States,^4^ and several European countries,^5^ yet the determinants of adverse birth outcomes among immigrants are not well understood. These include pre- and post-migration exposures that are not shared by native-born women (e.g. country of origin influences, language competence, and cultural assimilation after arrival).^6^–^11^ Duration of residence after arrival has been identified as a major predictor of health outcomes among immigrants,^12^–^15^ but this aspect has been rarely studied in perinatal health.^16^ Much of the research on the effects of migration on perinatal outcomes has compared births to first- versus second-generation women,^16^,^17^ but very few studies have assessed changes in birth outcomes among first-generation migrants.^18^–^20^

The assessment of health disparities by immigrants’ duration of residence involves two relevant comparisons. First, birth outcomes of immigrant subgroups defined by time in the receiving country can be compared with those of native-born women. Such a comparison allows the assessment of whether any disparities observed between newly arrived immigrants and non-immigrants change with increasing time spent in the new country. One common assumption in the literature is provided by the ‘convergence hypothesis’, inspired by the studies on coronary heart disease and stroke gradients of Japanese men living in Japan, Hawaii, and California.^21^–^23^ The convergence hypothesis suggests that health outcomes of immigrants will tend to converge over time towards the level observed in the host population, presumably via changes in health-related behaviours.^21^

The second comparison is among immigrants, and assesses whether health outcomes of recent immigrants differ from those with a longer time of residence. This comparison may provide clues about environmental influences, and may be relevant from a surveillance standpoint.

Comparisons by duration of residence may also help clarify other hypotheses regarding immigration and health, such as the so-called ‘epidemiologic paradox’ and the ‘healthy migrant effect’. The epidemiologic or ‘Hispanic paradox’ refers to the findings that Mexicans in the USA tend to have lower rates of low birthweight than USA-born women, despite their lower socioeconomic status.^24^–^26^ It has been suggested that studies on the Hispanic paradox of low birthweight should focus on PTB rather than on birthweight.^27^ It is increasingly recognised that low birthweight is not an optimal outcome for epidemiologic studies, as it may result from either short gestation or impaired fetal growth, without discriminating between the two. The ‘healthy migrant effect’ refers to favourable outcomes for immigrants, apparently as a result of the selection processes characterising migration flows, although such a healthy effect may not apply to immigrants from some regions of the world, such as South Asians.^17^ These two hypotheses have largely been studied ignoring the contribution of duration of residence. If duration of residence is associated with birth outcomes, then the epidemiologic paradox and the healthy migrant effect may only apply to certain types of immigrants (e.g. recent immigrants), but not to all.

Our first objective was to test the convergence hypothesis for PTB and small-for-gestational-age (SGA) birth in urban Ontario: that is, to compare immigrant subgroups defined by their duration of residence versus non-immigrants.

Our second objective, restricted to immigrants only, was to examine changes in the occurrence of PTB and SGA birth according to maternal duration of residence after arrival.

Urban Ontario is an appropriate setting to study the health of immigrants as Ontario is Canada’s most populous province, and annually receives about half of all immigrants arriving in Canada (approximately 120 000 each year), with more than 90% of them concentrated in urban areas.^28^

## Methods

### Study design

This is a population-based, data-linkage, cross-sectional study with retrospective assessment of immigration characteristics. Outcomes, and some maternal and obstetric characteristics, were obtained from hospital records within the most recent 5-year period (April 2002–March 2007), in order to ensure that their measurement was the same for all of the study subjects, and not affected by secular trends in the outcomes or by changes in coding schemes and reporting practices over time. Duration of residence and other immigration characteristics were retrospectively assessed via a data linkage with an immigration database (January 1985–December 2000), mostly built on legal documentation provided by the immigrants during their application processes.

### Data sources

The Discharge Abstract Database (DAD) of the Canadian Institute for Health Information (CIHI) compiles information on admissions, services, and discharges of all acute Ontario hospitals, where approximately 99% of provincial deliveries take place, excepting home births. The DAD is an excellent source for population-based estimates of perinatal outcomes.^29^–^31^ Birth and maternal records were internally merged according to an algorithm based on infant birth date, maternal admission and discharge dates, maternal and infant diagnoses and obstetric procedures, institution, and postal codes. Ninety-six percent of mothers were thus matched to an infant in the period April 2002–March 2007. Data on immigration were contained in the Landed Immigrant Data System (LIDS), which is the official immigration registry compiled by Citizenship and Immigration Canada (CIC). The LIDS contains sociodemographic and immigration characteristics that are not available for non-immigrants. These records were linked with hospital birth data via a linkage with the registry of the Ontario Health Insurance Plan (OHIP), which provides universal access to physician and hospital services to all Ontario residents (except for asylum seekers and immigrants during the first 3 months of residence). This linkage matched 84% of all immigrants whose intended destination was Ontario, and who obtained their legal permanent residence from 1 January 1985 to 31 December 2000. Immigration to Canada is regulated by a points system rewarding working age individuals who can successfully join the labour market. Since 1985 the official integration model for immigrants has been multiculturalism, characterised by policies aiming to counter discrimination, combat racism, and ensure equal opportunities in all areas. Many non-matched individuals may have moved outside Ontario after their arrival, and the remainder may be misclassified as non-immigrants. These data were finally merged with small-area data (census tracts) from the 2001 Canadian census.

### Study population

We extracted all 474 614 singleton live births born to mothers having a valid provincial health-card number (to ensure that they were Ontario residents) and living in any of the 11 Ontario Census Metropolitan Areas (Great Sudbury, Hamilton, Kingston, Kitchener, London, Oshawa, St. Catherines-Niagara, Ottawa-Gatineau, Thunder Bay, Toronto, and Windsor)^32^ at the time of delivery, between 1 April 2002 and 31 March 2007. In order to avoid misclassification of immigrant status regarding immigrants obtaining their permanent residence after December 2000, we excluded 74 961 infants whose mothers were first registered into the OHIP after 31 March 2001 (to account for the 3-month waiting period), who may be newcomers either from abroad or from other provinces. We further excluded the following categories of births: births weighing less than 500 grams and more than 6000 grams (*n* = 360), because of their high likelihood of being data errors; births with missing information on birthweight or gestational age (*n* = 125); births with gestational ages of less than 22 and more than 43 completed weeks (*n*= 72); births with missing information on infant sex, parity, or maternal age (*n*= 54); births with missing information on maternal sociodemographic and immigration characteristics (*n*= 576); and births to immigrants classified as ‘other’ (*n*= 487). After excluding 509 records to which census information could not be assigned, our study population for analyses comprised 397 470 singleton live births (with 83 233 born to immigrant mothers).

### Outcomes

All outcomes were categorised into binary variables. Preterm birth was defined as a delivery before 37 weeks of gestation completed, and was divided into preterm subgroups (<28 weeks, 28–31 weeks, 32–33 weeks, and 34–36 weeks). In Ontario, gestational age is largely estimated by ultrasound dating, and since 2002 hospital medical records departments have recorded gestational age based on the attending physician’s best interpretation of all clinical data, backed up by documentation from nursing staff as a secondary source.^33^ Small for gestational age (SGA) was defined as a birthweight below the tenth percentile of the most recent Canadian sex- and gestational age-specific birthweight distribution.^34^

### Predictors

Mothers were categorised as foreign-born if listed in the immigration database. Non-immigrants included Canadian-born women and some immigrant women with a residency of longer than 17 years (i.e. permanent residence obtained before 1985). As information on sociodemographic characteristics was only available from the immigration data, we could not measure these characteristics among the non-immigrant group. Information from hospital records available for the entire study population was measured at the time of delivery/birth, and included infant sex (male versus female), maternal age at delivery (15–19, 20–24, 25–29, 35–39, ≥40 years versus 30–34), and parity (primiparae versus multiparae).

Information specific to immigrants was obtained from their landing records, last updated at the port of entry on the date of their arrival to Canada. With the exception of language knowledge, which was self-reported, the remaining information was ascertained based on legal documentation provided by the immigrants during their application processes. We assessed the level of exposure to the Canadian setting by duration of residence in Canada as completed years from arrival to delivery. We also modelled duration in approximately 5-year duration groups (15 months to 4 years, 5–9 years, 10–14 years, and 15 years or more).

Relevant covariates included maternal country of birth, high school graduation (no versus yes), marital status (single, widowed, or separated versus married or common law), immigrant class (economic class, refugee status versus family class), and knowledge of either of the official Canadian languages (none versus English or French). Countries of birth were grouped into world regions using a modified version of the UNICEF classification, with industrialised countries as the reference group (Appendix S1).^35^ We excluded maternal age at arrival and infant gender because they were not associated directly or indirectly with either the exposure or the outcome. Maternal morbidity during pregnancy was ascertained using the International Statistical Classification of Diseases and Related Health Problems, Tenth Revision, Canada (ICD-10-CA), and included genito-urinary infections (ICD-10-CA: O230–O235, O239), pregnancy-induced hypertension (O13, O140, O141, O149), gestational diabetes (O24.4), incompetent cervix (O343), and placental abruption (O450, O458, O459). Obstetric procedures were identified through the Canadian Classification of Interventions (CCI), and included induction of labour (CCI: 5.AC.30) and caesarean section (5.MD.60). Maternal morbidity and obstetric procedures were considered as mediating the effect of duration of residence on PTB, and therefore were not included in the final models. We also assigned each mother a neighbourhood deprivation score developed for Canadian urban settings,^36^ but this variable was not included in the final models because it mediates the effect of country of origin on birth outcomes via self-selection of immigrants into particular neighbourhoods (i.e. immigrants from poor countries tended to settle into poor neighbourhoods, and vice versa).^37^

### Statistical analyses

We inspected the shape of the relationship between duration of residence and adverse birth outcomes using bivariate plots and logistic models with linear, quadratic, and cubic terms, as well as with dummy variables. A linear specification of duration resulted in a better fit, as assessed by the likelihood ratio test.

To compare outcomes of all immigrants and immigrant subgroups versus non-immigrants we used logistic regression to compute odds ratios with 95% confidence intervals, adjusted for maternal age and parity at delivery.

Analyses restricted to immigrants only were more complex because of the availability of richer information. To account for the clustering of births within maternal countries of birth among immigrants, we modelled countries as two-level random intercepts. Multilevel logistic regression analyses using GLIMMIX in sas 9.1 (SAS Institute, Cary, NC, USA) were used to estimate the effects of duration of residence on the outcomes, with 5-year adjusted odds ratios with 95% confidence intervals. We also calculated the predicted probabilities of PTB according to length of residence in Canada. Finally, to assess whether the association between duration of residence and birth outcomes varied according to the maternal region of birth, we tested for interaction by including a product term (duration × region of birth) in the adjusted models.

### Sensitivity analyses

We repeated multivariate analyses with the excluded variables infant sex, maternal morbidity during pregnancy, induction of labour, caesarean section, and neighbourhood deprivation. We further adjusted for year of arrival to evaluate the possibility that the observed associations between duration of residence and adverse birth outcomes were confounded by cohort effects. We also adjusted for the infant’s year of birth to eliminate any residual confounding resulting from trends in PTB within the study period. Finally, we restricted analyses to immigrant women who arrived at age 20 or older to reduce the misclassification of maternal education and marital status.

## Results

Immigrants differed from non-immigrants in the outcomes and in most covariates (Table 1). Immigrants exhibited higher proportions of PTB with increasing duration of residence, but not of SGA. Among immigrants, marital status, education, and knowledge of official languages differed according to duration of residence, but some of these differences resulted from the fact that these variables were measured on arrival, and were therefore affected by the maternal age at arrival. Yet, the trends across duration groups remained significant after restricting the population to women aged 20 years or older at arrival (not shown). Higher proportions of refugees and immigrants from Latin America and industrialised countries with more years of residence reflect the changing immigration patterns over time, more recently dominated by immigrants from East and South Asia.

**Table 1:** Characteristics of the study population of singleton live births in the period 2002–2007 in urban Ontario, by migrant status and immigrants’ duration of residence*

	Non-immigrants	All immigrants	*P*-value**	Immigrants by duration of residence				
				<5 years	5–9 years	10–14 years	≥15 years	*P* trend***
**Number of births**	314 237	83 233		14 555	32 539	23 827	12 312	
**Outcomes (weeks)**								
<37	6.3	6.0	0.0059	4.7	5.6	6.6	7.4	<0.0001
<28	0.3	0.4	<0.0001	0.3	0.4	0.5	0.6	<0.0001
28–31	0.5	0.6	0.0548	0.4	0.5	0.6	0.7	<0.0001
32–33	0.7	0.7	0.2442	0.5	0.6	0.8	0.9	<0.0001
34–36	4.8	4.3	<0.0001	3.6	4.1	4.7	5.2	<0.0001
Small for gestational age	7.7	11.1	<0.0001	11.4	11.0	11.0	11.0	0.3145
**Infant and maternal characteristics**								
Infant sex (male)	51.2	51.7	0.0149	51.7	51.8	51.3	51.9	0.9409
**Maternal age at delivery (years)**								
<20	3.7	1.8	<0.0001	1.1	1.6	2.5	2.0	<0.0001
20–24	11.4	10.9	<0.0001	12.9	9.7	10.8	11.8	0.2868
25–29	26.2	27.9	<0.0001	33.5	28.7	23.9	27.0	<0.0001
30–34	36.5	34.0	<0.0001	35.7	36.7	31.7	29.4	<0.0001
35–39	18.5	20.5	<0.0001	14.7	19.7	24.5	21.8	<0.0001
≥40	3.6	4.9	<0.0001	2.3	3.6	6.6	8.1	<0.0001
**Parity at delivery (no previous live birth)**	46.5	36.6	<0.0001	41.0	33.7	35.8	40.2	0.5541
**Unmarried/not cohabiting at arrival**		56.1		18.4	45.6	76.2	89.4	<0.0001
**No high school graduation at arrival**		63.4		38.2	54.7	77.5	88.7	<0.0001
**Knowledge of English/French at arrival**		56.6		51.9	55.6	58.5	61.0	<0.0001
**Immigrant class at arrival**								
Economic		28.7		38.7	27.0	23.0	32.5	<0.0001
Family		57.4		57.3	64.0	56.9	41.1	<0.0001
Refugee		13.9		4.0	9.0	20.1	26.4	<0.0001
**World region of birth**								
Central/East Europe		7.4		9.3	9.6	5.7	2.8	<0.0001
Caribbean		12.7		3.7	9.1	17.3	24.1	<0.0001
Hispanic America		5.5		3.5	3.7	6.2	11.3	<0.0001
Middle East/North Africa		7.4		9.2	8.2	6.3	5.3	<0.0001
East Asia/Pacific		18.3		22.5	19.3	17.1	13.2	<0.0001
South Asia		27.1		40.2	32.4	19.9	11.4	<0.0001
Sub Saharan Africa		7.7		4.2	7.6	10.6	6.6	<0.0001
Industrialised Countries		13.9		7.5	10.6	17.0	25.4	<0.0001

* Cell entries are percentages, unless otherwise specified.

** Chi-square test for the difference of proportions between immigrants and non-immigrants.

*** Two-sided *P*-value of Cochran-Armitage test for trends in binomial proportions across duration groups (when the variable had more than two categories, each category was compared with the remainder).

### Immigrants compared with non-immigrants

Compared with non-immigrants, all immigrants, as a single category, had similar odds of PTB (<37 weeks) (Table 2). When immigrants were categorised by duration, recent immigrants with less than 10 years of residence had lower odds of PTB than their Canadian counterparts, but this reversed among those with 10 or more years of residence. The duration of stay at which immigrants became at higher risk than non-immigrants, however, varied across preterm subgroups: among extremely preterm infants (<28 weeks), it happened after 5 years; among infants born within 28–33 weeks of gestation, it happened after 10 years; and among those born at 34–36 weeks of gestation it happened after 15 years. All immigrants were also at higher odds of SGA than non-immigrants, and the association did not vary across duration groups.

**Table 2:** Odds ratios (with 95% confidence intervals) for adverse birth outcomes between immigrants and non-immigrants overall, and by duration of residence, in live births during 2002–2007 in urban Ontario

	Preterm	Preterm subgroups				Small for gestational age
	<37 weeks	<28 weeks	28–31 weeks	32–33 weeks	34–36 weeks	OR* (95% CI)
	OR* (95% CI)	OR* (95% CI)	OR* (95% CI)	OR* (95% CI)	OR* (95% CI)	
**Non-immigrants**	1.00	1.00	1.00	1.00	1.00	1.00
**All immigrants**	0.98 (0.95–1.01)	1.63 (1.44–1.84)	1.15 (1.03–1.27)	0.98 (0.89–1.07)	0.92 (0.89–0.96)	1.58 (1.54–1.62)
**Immigrants by duration (years)**						
<5	0.77 (0.71–0.83)	0.96 (0.69–1.34)	0.86 (0.67–1.12)	0.69 (0.54–0.88)	0.76 (0.70–0.83)	1.60 (1.52–1.69)
5–9	0.92 (0.87–0.97)	1.60 (1.33–1.91)	1.04 (0.88–1.22)	0.84 (0.72–0.98)	0.88 (0.83–0.93)	1.60 (1.54–1.66)
10–14	1.07 (1.01–1.13)	1.77 (1.46–2.16)	1.29 (1.09–1.53)	1.21 (1.04–1.40)	0.98 (0.92–1.04)	1.56 (1.50–1.63)
≥15	1.20 (1.12–1.28)	2.18 (1.72–2.77)	1.47 (1.18–1.82)	1.22 (1.00–1.48)	1.10 (1.01–1.19)	1.52 (1.43–1.61)

* Odds ratio adjusted for maternal age and parity at delivery.

**Figure 1:**
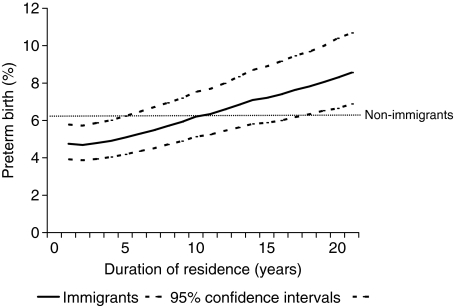
Preterm birth percentages, adjusted for maternal age at delivery, parity, immigrant class, country and region of birth, language knowledge on arrival, high school graduation on arrival, unmarried status on arrival, with 95% CI, among immigrants by years spent in urban Ontario, and non-immigrants, for all singleton live births in the period 2002–2007.

Figure 1 depicts the PTB rates based on a two-level model, with births nested within maternal countries of birth, including duration of residence as a continuous variable, and adjusted for the covariates listed in Table 3. The flat line represents the crude PTB rate (<37 weeks) of non-immigrants in the study period (Table 1). Recent immigrants (<5 years) compared favourably versus non-immigrants, but this advantage was lost with 10 years of residence, and long-term immigrants (≥15 years) experienced the highest risk.

**Table 3:** Adjusted odds ratios (with 95% confidence intervals) for adverse birth outcomes in live births during 2002–2007 in urban Ontario, by immigrants’ duration of residence

	Preterm	Preterm subgroups				Small for gestational age
	<37 weeks	<28 weeks	28–31 weeks	32–33 weeks	34–36 weeks	OR* (95% CI)
	OR* (95% CI)	OR* (95% CI)	OR* (95% CI)	OR* (95% CI)	OR* (95% CI)	
**Duration of residence (years)**						
5-year OR*	1.14 (1.10–1.19)	1.23 (1.07–1.42)	1.14 (1.00–1.29)	1.16 (1.03–1.30)	1.13 (1.08–1.19)	0.99 (0.96–1.02)
<5	1.00	1.00	1.00	1.00	1.00	1.00
5–9	1.14 (1.04–1.26)	1.54 (1.05–2.25)	1.12 (0.82–1.54)	1.08 (0.81–1.45)	1.13 (1.01–1.26)	0.98 (0.92–1.05)
10–14	1.27 (1.14–1.42)	1.58 (1.03–2.42)	1.30 (0.91–1.84)	1.42 (1.03–1.96)	1.22 (1.08–1.38)	0.97 (0.90–1.05)
≥15	1.39 (1.23–1.58)	1.88 (1.17–3.02)	1.43 (0.96–2.13)	1.41 (0.97–2.04)	1.35 (1.16–1.56)	0.95 (0.86–1.05)
***P* trend**	<0.001	0.026	0.052	0.019	<0.001	0.299
**Maternal region of birth**						
Industrialised countries	1.00	1.00	1.00	1.00	1.00	1.00
Central & Eastern Europe	1.01 (0.81–1.26)	1.52 (0.82–2.83)	1.00 (0.60–1.68)	0.87 (0.52–1.45)	1.02 (0.81–1.29)	0.76 (0.60–0.95)
Middle East & North Africa	0.96 (0.77–1.21)	0.90 (0.44–1.86)	1.12 (0.68–1.85)	0.53 (0.29–0.96)	1.00 (0.79–1.26)	1.12 (0.90–1.39)
Sub-Saharan Africa	1.25 (1.00–1.56)	2.53 (1.43–4.49)	1.11 (0.67–1.82)	1.33 (0.83–2.13)	1.10 (0.87–1.39)	1.33 (1.07–1.65)
Caribbean	1.72 (1.37–2.17)	2.94 (1.67–5.18)	2.06 (1.34–3.16)	1.91 (1.22–2.98)	1.55 (1.22–1.96)	1.80 (1.42–2.28)
Hispanic America	1.13 (0.91–1.41)	1.45 (0.75–2.78)	1.04 (0.61–1.79)	1.35 (0.85–2.26)	1.10 (0.87–1.38)	0.96 (0.77–1.19)
East Asia & Pacific	1.30 (1.02–1.64)	0.97 (0.52–1.80)	1.18 (0.77–1.83)	1.06 (0.66–1.68)	1.34 (1.06–1.70)	1.43 (1.12–1.82)
South Asia	1.26 (0.99–1.61)	1.78 (1.00–3.15)	1.42 (0.93–2.17)	1.11 (0.71–1.75)	1.25 (0.98–1.59)	1.79 (1.38–2.33)

* Odds ratio adjusted for maternal age at delivery, parity at delivery, immigrant class, country and region of birth, language knowledge on arrival, high school graduation on arrival, and unmarried status on arrival.

### Internal comparisons between immigrants by duration of residence

Duration of residence was independently associated with PTB and all preterm subgroups in multilevel models further adjusted for maternal sociodemographic and immigration characteristics, although duration did not reach statistical significance for infants born within 28–31 weeks of gestation (Table 3). Although the 5-year odds ratios were somewhat higher for early deliveries (<28 weeks), the confidence intervals overlapped. Compared with women from industrialised countries, women from the Caribbean were more likely to deliver preterm babies as gestational age decreased. Sub-Saharan Africans were also at higher odds of extreme PTB (<28 weeks). Duration of residence was not associated with SGA, but maternal region of birth was.

Finally, an interaction between duration and maternal region of birth was not significant for all outcomes. Stratified analyses by maternal region of birth were consistent, and showed increases in PTB with increasing duration of stay for all regions of the world.

### Sensitivity analyses

Although we based our covariate adjustment on theoretical considerations, we also fitted the models with the excluded variables maternal morbidity during pregnancy, induction of labour, caesarean section, neighbourhood deprivation, and infant sex, but the results of Table 3 remained unaffected. The addition of the mother’s year of arrival or infant’s year of birth did not substantially change the estimates either. As maternal education, marital status, and knowledge of official languages were measured on arrival, and were therefore affected by the age at arrival, we repeated our analyses restricted to women aged 20 years or older on arrival, but the association between duration of residence and PTB did not substantially change (not shown). In *post hoc* analyses restricted to preterm infants we did not find statistically significant trends in induction of labour (*P*= 0.187) or caesarean sections (*P*= 0.074) with duration of residence.

## Discussion

### Main findings

Our findings indicate that the duration of residence of immigrants in urban Ontario was independently associated with increases in PTB, but not in SGA. Recent immigrants were at lower risk of PTB compared with a mostly Canadian-born population, but immigrants became at higher risk after 10 years of stay in Canada. There was no strong evidence indicating that the influence of duration of residence on birth outcomes varied with the maternal region of birth or with preterm subgroups.

### Limitations

Our study had some limitations. Non-matched immigrants who stayed in Ontario after arrival were counted as non-immigrants, thus diluting the differences in outcomes between these two groups. Because data collection on immigrants started in 1985, we could not identify those immigrants who obtained their permanent residency before 1985. This misclassification, however, would bias our results against significance, because false non-immigrants were immigrants with at least 17 years of residence, and so were at a higher risk of PTB. Because immigration data were linked until 31 December 2000, we had to exclude all newcomers to Ontario after that date to avoid misclassification by immigrant status. This resulted in some under-representation of recent immigrants in our study population. Although the immigration data were of good quality, some variables were measured on arrival and not at delivery, which is a concern for time-dependent variables. Marital status and maternal education may have changed for some women, especially for young women on arrival, who may have got married and become more educated with a longer duration of residence. As educational attainment is not reversible, the bias resulting from adjustment for an underestimate of the true educational attainment of long-term immigrants would have reduced the significance of our results, because higher education protects against adverse birth outcomes. To reduce this bias, we repeated analyses restricted to mothers aged 20 years or older on arrival, but the results did not substantially change.

The use of a Canadian standard for SGA^34^ may not be appropriate for some ethnic groups (e.g. Asians), for which lower birthweight for gestational age is likely to reflect physiological rather than pathological differences.^38^,^39^ However, even if the use of a non ethnic-specific standard overestimates the association between immigrant status and SGA, it is less likely to affect the association between duration of residence and SGA, as the measurement of SGA was independent of duration of residence.

Finally, we had no data on some important predictors of the outcomes, such as tobacco smoking, alcohol consumption, and maternal height, weight, or body mass index (BMI). However, these are mediators rather than confounders of the association between duration of residence and birth outcomes.

### Interpretation

Despite these limitations, the association between duration of residence and PTB was quite robust, remaining unaltered across a range of sensitivity analyses. Our main finding regarding a linear increase in PTB with time spent in Canada is consistent with previous studies on Mexicans showing increases in PTB after 2 years of residence in Washington,^18^ and after 5 years of residence in California.^19^ Unlike the study that found that Finns lowered their risk of low birthweight and PTB after 3 years of stay in Sweden, but that Sub-Saharan Africans did not,^20^ we did not find evidence that the association between duration of residence and PTB varied by the maternal world region of origin. Although the increase in PTB with time spent in Canada affected all women, it is noteworthy that a significant number of immigrant women were children or adolescents on arrival. This suggests that adolescent immigrants may constitute a vulnerable cohort, because this is the group that accounts for most deliveries 10–20 years later.

The increase in PTB rates observed in the USA and Canada during the last two decades has been mainly attributed to increased obstetric interventions near term to prevent neonatal complications.^3^,^40^ We did not find evidence that this trend observed in the whole population during the last decade was mirrored among immigrants after arrival, suggesting that a ‘population health’ perspective^41^ may be more appropriate than medical care patterns to explain changes in PTB with time spent in Canada.

Our findings do not support the ‘convergence hypothesis’, which seems to hold for increased BMI/obesity^13^,^14^ and behavioural risk factors,^12^,^14^,^15^ and is not consistent for mortality.^21^ Recent immigrants were at lower risk of PTB, but lost their advantage after approximately 10 years. Instead of remaining at that level, as predicted by the hypothesis, immigrants experienced a continuous deterioration that placed them at a disadvantage after 10 years of stay, compared with non-immigrants. Moreover, because SGA was higher among recent immigrants than among non-immigrants, convergence would have predicted a decrease over time, but duration of residence had no visible impact on this outcome. The definition of the comparison groups and the length of the observation period may impact on the study conclusions. Our study is unique in its ability to measure durations of residence of over 20 years. If our data had been limited to immigrants with less than 10 years of stay we would have erroneously concluded there was convergence in PTB, and missed the observed ‘overshoot’ after 10 years of stay.

Regarding the frequently debated epidemiologic paradox of low birthweight and PTB and the healthy migrant effect, it is noteworthy that these two hypotheses have been largely discussed in the literature in the absence of information on duration of residence. Our findings on PTB suggest that these two phenomena may apply only to recent immigrants. Although studies reporting the epidemiologic paradox in PTB could not distinguish between recent and long-term immigrants, it is likely that the results were driven by the healthier recent immigrants, as most immigrant women have their babies within 10 years of migration. Recent immigrants may enjoy better health than the native-born women soon after arrival, but lose the short-term benefits of selective migration with cumulative exposure to the new physical and social environment, experiencing a sort of ‘regression to the mean’.^42^

The lack of an association between duration and SGA births in our study suggests that the influence of duration of residence on perinatal health may be outcome-specific. Although our data were not detailed enough to elucidate mechanisms explaining the deterioration of PTB among immigrants, we can advance some hypotheses. One candidate explanation is acculturation, which has already been linked with adverse birth outcomes.^7^,^8^,^10^,^43^ This suggests that the effect of duration of residence might be explained by changes in health behaviours and related risk factors. Indeed, duration of residence has been used as a proxy for acculturation, and has been associated with increases in BMI/obesity, smoking, alcohol consumption, and physical inactivity,^12^–^15^,^44^,^45^ factors that may negatively affect gestational age. The prevalence of these factors among immigrants to Canada has been reported to increase with longer stays in Canada.^46^–^49^ Whereas high pre-pregnancy BMI has been associated with increases in at least one PTB subtype in some studies,^50^–^53^ it has been more consistently found to be protective for SGA.^50^,^51^,^54^,^55^ It is reasonable to speculate that in our immigrant population increases in BMI might have compensated for the detrimental effect of changes in other risk factors affecting gestational age, thus rendering a null association between duration of residence and SGA.

Another potential pathway leading to an association between duration of residence and PTB, but not SGA, albeit unexplored among immigrant women, may involve psychosocial factors. A few studies found that different measures of maternal stress were associated with PTB, but not with intrauterine growth restriction.^56^–^58^ Working conditions, such as long working hours, prolonged standing, and physically demanding work has also been associated with PTB.^59^–^61^ Psychosocial exposures such as job strain or low job control and satisfaction may be concomitant causes of PTB among immigrant women,^59^,^60^,^62^–^64^ who are more likely to be employed in manual, clerical, and shift jobs, and in jobs requiring less than their education level.^65^

Previous studies have described increases in PTB rates among Mexicans with prolonged residency in the USA. This study was conducted on an ethnically diverse immigrant population, and the association between duration and PTB did not vary according to the world region of maternal origin. This suggests that not only Hispanics but all immigrants were equally affected by prolonged residency in urban Ontario. However, our findings cannot be generalised to rural areas, urban settings with distinctive immigrant groups, or to other health outcomes, without additional empirical research.

## Conclusion

Immigrants’ duration of residence was associated with increases in the risk of preterm delivery, but not SGA births. This suggests the presence of outcome-specific processes related to duration of residence. The fact that important changes in preterm delivery take place within relatively short periods of time after migration suggests that their causes are mainly environmental, and therefore at least theoretically preventable. Ignoring duration of residence may mask important disparities in preterm delivery between immigrants and non-immigrants, and between immigrant subgroups. The clarification of the mechanisms behind the association between duration of residence and PTB merits further investigation.

### Disclosure of interests

The authors declare that they have no competing interests.

### Contribution to authorship

MU conceived the study, performed the analyses, and led the writing of the manuscript. JF and RG supervised the study, helped to interpret the results, and revised the manuscript for important intellectual content. RM helped with the statistical methods and helped interpret the results. All authors read and approved the final version of the manuscript.

### Details of ethics approval

The use of the data was approved by the Research Ethics Boards of the Sunnybrook Health Sciences Centre and the University of Toronto (protocol reference #23550), Toronto, Ontario.

### Funding

No funding was sought or obtained to undertake this specific study. MU was supported by a personal research grant (CIHR IOP-44972) from JF, Canadian Institutes of Health Research.

## Supporting information

The following supplementary materials are available for this article:

**Appendix S1.** Country classification.
