# Supplementary material for: Immigrants’ duration of residence and adverse birth outcomes: a population-based study
Source: BJOG. 2010 Apr;117(5):591–601. doi: 10.1111/j.1471-0528.2010.02523.x (PMC2848981; doi:10.1111/j.1471-0528.2010.02523.x)
Supplement: Supplementary file 1 [file bjo0117-0591-SD1.doc]

**Appendix S1: Country classification**

**Industrialized countries**

Andorra

Australia

Austria

Belgium

Canada

Czech Republic

Denmark

Estonia

Finland

France

Germany

Greece

Hong Kong, China

Hungary

Iceland

Ireland

Israel

Italy

Japan

Latvia

Lithuania

Luxembourg

Netherlands

New Zealand

Norway

Poland

Portugal

Republic of Korea

San Marino

Slovakia

Slovenia

Spain

Sweden

Switzerland

Turkey

United Kingdom

United States of America

**Central & East Europe**

Albania

Armenia

Azerbaijan

Belarus

Bosnia and Herzegovina

Bulgaria

Croatia

Georgia

Kazakhstan

Kyrgyzstan

Macedonia (The former Yugoslav Republic of)

Moldova

Montenegro

Romania

Russian Federation

Serbia

Tajikistan

Turkey

Turkmenistan

Ukraine

Uzbekistan

**Middle East & North Africa**

Algeria

Bahrain

Djibouti

Egypt

Iran (Islamic Republic of)

Iraq

Jordan

Kuwait

Lebanon

Libyan Arab Jamahiriya

Morocco

Occupied Palestinian territory

Oman

Qatar

Saudi Arabia

Sudan

Syrian Arab Republic

Tunisia

United Arab Emirates

Yemen

**Sub Saharan Africa**

*Eastern and Southern Africa*

Angola

Botswana

Burundi

Comoros

Eritrea

Ethiopia

Kenya

Lesotho

Madagascar

Malawi

Mozambique

Namibia

Rwanda

Seychelles

Somalia

South Africa

Swaziland

Tanzania, United Republic of

Uganda

Zambia

Zimbabwe

*West and Central Africa*

Benin

Burkina Faso

Cameroon

Cape Verde

Central African Republic

Chad

Congo

Congo, Democratic Republic of the

Côte d'Ivoire

Equatorial Guinea

Gabon

Gambia

Ghana

Guinea

Guinea-Bissau

Liberia

Mali

Mauritania

Niger

Nigeria

Sao Tome and Principe

Senegal

Sierra Leone

Togo

**Caribbean**

Antigua and Barbuda

Barbados

Belize

British Virgin Islands

Dominica

Grenada

Guyana

Haiti

Jamaica

Montserrat

Saint Kitts and Nevis

Saint Lucia

Saint Vincent and the Grenadines

Suriname

Trinidad and Tobago

Turks and Caicos Islands

**Hispanic America**

Argentina

Bolivia

Brazil

Chile

Colombia

Costa Rica

Cuba

Dominican Republic

Ecuador

El Salvador

Guatemala

Honduras

Mexico

Nicaragua

Panama

Paraguay

Peru

Uruguay

Venezuela (Bolivarian Republic of)

**East Asia & Pacific**

Cambodia

China

Cook Islands

Fiji

Indonesia

Kiribati

Korea, Democratic People's Republic of

Lao People's Democratic Republic

Malaysia

Marshall Islands

Micronesia (Federated States of)

Mongolia Myanmar

Nauru

Niue

Palau

Papua New Guinea

Philippines

Samoa

Solomon Islands

Thailand

Timor-Leste

Tokelau

Tonga

Tuvalu

Vanuatu

Viet Nam

**South Asia**

Afghanistan

Bangladesh

Bhutan

India

Maldives

Nepal

Pakistan

Sri Lanka
